# Reviewing retrieved references for inclusion in systematic reviews using EndNote

**DOI:** 10.5195/jmla.2017.111

**Published:** 2017-01

**Authors:** Wichor M. Bramer, Jelena Milic, Frans Mast

An important task in conducting a systematic review is reading titles and abstracts of the retrieved references, which often number in the thousands, to determine which articles meet the predefined inclusion criteria. In the past, this was performed by manually scanning through large stacks of printed titles and abstracts, followed by face-to-face meetings to discuss which references should be included. Today, the workflow of the review process is more streamlined by using computer software.

Several specialized solutions for this process are available, most notably free or subscription-based online tools such as Covidence, DistillerSR, or Rayyan. The Cochrane collaboration uses its own tool called ReviewManager. A survey in 2013 showed that more than half of all systematic reviewers used EndNote software [1]. This dominance on the market is likely to increase as sales of the second most popular tool, Reference Manager, have ceased, and its website now advises users to switch to EndNote. Many reviewers use Microsoft Excel. Some libraries have even created specialized Excel workbooks to document the process in much detail [2]. A method for the inclusion process using EndNote is described by King et al. [3], but this process is rather complicated and time-consuming.

This paper describes the logistics of a method to perform the title and abstract screening, verdict assignment, and comparisons of results among multiple reviewers in EndNote. The process is blinded; all reviewers work in their own EndNote files, and after the individual inclusion and exclusion processes, the verdicts of the different reviewers are compared. The method described here can be performed much faster than comparable methods.

## THE METHOD

The method consists of several steps. First, a custom style should be installed for easy abstract scanning. Second, a field should be added to show the reviewer’s name in the Library window. Third, for each systematic review, custom groups are made in the EndNote library for included and excluded references. Reviewers drag articles to the group corresponding to their verdicts. In the last steps for the final comparison of the verdicts, the included references of all reviewers are combined into one EndNote library and de-duplicated. References found as duplicates are included by both reviewers and are selected for full-text review, the non-duplicate references can then be discussed for inclusion or exclusion.

The steps below are written for EndNote X7 for Windows. EndNote is also available in a version for Apple Mac computers, but some of the menu items will appear on different places. The authors have added footnotes that guide Mac users as much as possible.

### Step 1: Install the custom-made output style

A custom-made style (named _preview) facilitates easier reviewing of the titles and abstracts.

**Figure 1:**
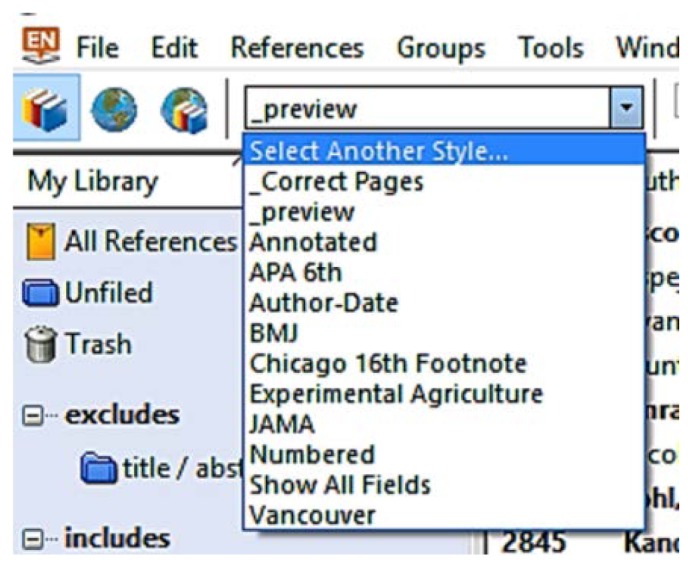
Activating the _preview style

1. Visit http://bit.ly/emcendnote
2. Open the zip file
3. Double-click on the file_preview.ens (it will open in EndNote)*
4. In EndNote, click File > Save As
5. Remove the text “copy” from the file name, and click Save†
6. Close the style using the cross in the top right corner‡
7. To activate the _preview style, open the drop-down menu (called Select Bibliographic Output Style) in the top left part of the screen (Figure 1)§
8. Click on Select another style, scroll to the top, select _preview, and click Choose
9. The abstract of the selected reference will be displayed in the preview tab in the Tab pane

### Step 2: Change the settings of the library window

When comparing the included references between reviewers, a special field will be used to document the name of the reviewer who included a certain reference. To be able to view this in the Library window, this field has to be added to the preference settings.

1. Go to Edit > Preferences > Display Fields
2. In column 8 (or an alternative field that is not often used), under Field, select Custom 4
3. In the same column, under Heading, type Reviewer

### Step 3: Create groups for inclusion and exclusion in the EndNote library containing the search results

**Figure 2:**
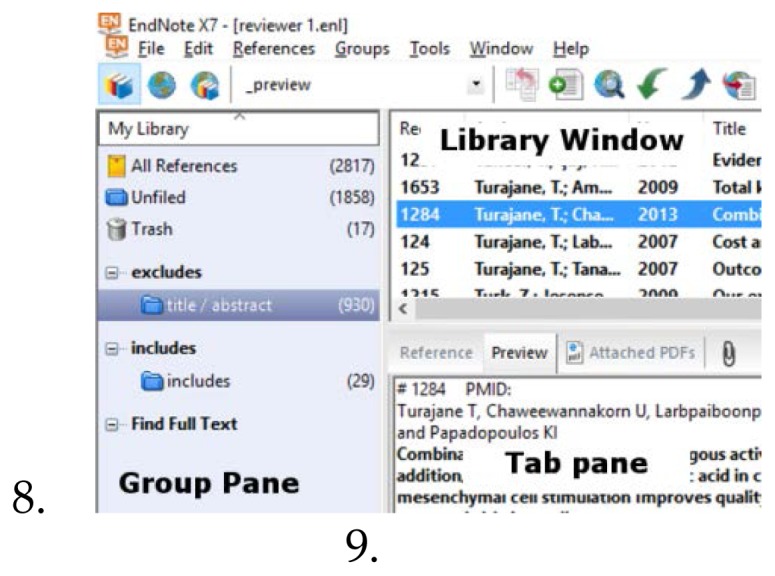
Groups for *Includes* and *Excludes*

In the first reviewing round, two reviewers independently read titles and abstracts to decide whether a reference is potentially relevant to the review. We propose the creation of two group sets: *Includes* and *Excludes,* each with, at this stage, just one subgroup (Figure 2).

1. Right-click on My Groups, and select Create Group Set
2. Type *Excludes,* and hit Enter
3. Right-click on *Excludes,* and select Create Group
4. Type *title/abstract,* and hit Enter
5. Repeat the process for Group Set *Includes* with *Includes* group

After the groups for inclusion and exclusion have been made, two copies of these files are created (adding the name of the reviewer to the file name) and distributed to the reviewers. All reviewers will work in their own copies of the EndNote file.

### Step 4: Title/abstract screening

The standard group named *Unfiled* contains references not yet assigned to one of the other groups. When starting the screening phase, the number of references in *Unfiled* will be equal to that in All References. Each reviewer reviews the relevance of references in *Unfiled* one by one, based on the title and/or abstract.

1. Broaden the title field in the Library window (Figure 2) by dragging the column break between the columns of Title and Journal to the right until the Title column reaches an appropriate width
2. Review the titles one by one in the Library window until a potentially relevant title is reached, without yet assigning references to the groups
3. Click on the relevant title, and read the abstract in the preview tab of the Tab pane (Figure 2)
4. If the abstract is irrelevant, continue reading titles in the Library window
5. If the abstract is relevant, select the article directly above that relevant article in the Library window, and press Ctrl+Shift+Home to select all references above it. Drag all of these articles into the Excludes > Title/Abstract group
6. Next, drag the top reference in the Library window (which is the reference to be included, as irrelevant references were removed from *Unfiled*) to the *Includes* > *Includes* group
7. Repeat this process until all references are filed and the *Unfiled* group is emptied

### Step 5: Compare included references between reviewers

1. Go to the folder *Includes* in the EndNote library of reviewer 1, and click on one of the references
2. Go to Tools > Change/Move/Copy Fields
3. In Custom 4, select Insert after field’s text, type the first name of reviewer 1, and click on OK in three pop-up screens
4. Open the EndNote library of reviewer 2 without closing that of reviewer 1
5. Go to the *Includes* group in the library of reviewer 2; select all references in that group, right click on one of them, select Copy reference to, and select the file screened by reviewer 1
6. Go to Window, and select the file by reviewer 1
7. Go to the *Unfiled* group, and mark the records in that group with the name of reviewer 2 (as described above in steps 2–3)
8. Drag the references from *Unfiled* into the *Includes* group
9. Check the settings for de-duplication (Edit > Preferences > Duplicates); at least Author, Year, Title, and Secondary Title (Journal) should be selected
10. Go to the group *Includes*, select a random reference, and go to References > Find Duplicates; in the detailed comparison screen, click Cancel; then press Delete on the keyboard to remove the duplicate references
11. Right click in the group set *Includes*, select Create a group, and name it *Definite Includes*
12. Select all references in the Duplicate References group, and drag those to the *Definite Includes* group
13. Go to the *Definite Includes* group, and mark the records in that group with the name of reviewer 2 (as described above in steps 2–3)
14. Right click on the *Includes* group > *Includes,* and select delete group
15. Have the two reviewers discuss the articles currently in the *Unfiled* group
16. After consensus is reached, drag the references one by one to the appropriate group until *Unfiled* is empty

### Step 6: Full-text reviewing

In the second round of screening, full texts of the included titles and abstracts need to be reviewed. Custom groups can be used to distinguish between various reasons for exclusion, and articles can be assigned to specific groups for certain sub-questions. All reviewers should again work in their own copies of this library. After reading all articles, each reference in the library should be discussed in detail; therefore, no automatic comparison should be used. The steps in this round are more laborious, differ per research topic, and can hardly be generalized and optimized. Therefore, we do not describe in detail how this process can be executed.

## DISCUSSION

We advise against the use of a “Doubt” or “Maybe” group for articles for which it is not yet clear whether they should be included. We recommend that in cases of doubt, the article should be added to the folder *Includes*. If the second reviewer also has doubts about the relevance or decided to include the article, the full text should be used for final judgment. If the second reviewer excludes the article, it is an item for discussion.

The process of reading titles and abstracts for inclusion and exclusion is often considered time consuming, and the number of abstracts that can be read per hour is estimated at 120 by the Cochrane Handbook [4]. A recent study recorded the time needed for certain steps in the systematic review process [5]. Upon request, the authors informed us that, using specialized screening software, the median number of articles that could be reviewed per hour was 68. Another recent study estimated the time needed to screen 1 record based on title and/or abstract at 1 minute [6]. A survey among review authors at Erasmus MC, to whom we had sent an earlier draft of this article, reported that the median number of titles and/or abstracts reviewed per hour with the present method was 308, with a maximum of 675.

We found that the speed of the process increases when reviewers do not document the specific reasons for excluding references during the title/abstract screening phase. It is often clear that an article is not relevant to the topic, but to determine the exact reason (or very often multiple reasons) is very time consuming and unnecessary. According to PRISMA guidelines, reasons for exclusion, with the number of articles for each reason, should be given only in the full-text screening phase [7]. We found that researchers who meticulously documented the reasons for exclusion in the first round and those who had used other software such as Microsoft Excel reported much slower rates (median of sixty minutes) and often later regretted their decision to do so. Also, using specialized programs or online systematic review management systems such as Covidence or DistillerSR unnecessarily complicate and delay the process because each abstract has to be assigned to categories individually, in contrast to this method’s bulk assignment of nonrelevant articles.

## Footnotes

* For Mac users: The file must be downloaded and saved locally before it can be opened.

† For Mac users: The text “copy” will not appear in the file name.

‡ For Mac users: The cross appears in the top left corner.

§ For Mac users: At the bottom right of the screen is a preview pane. The style selection menu appears at the top of the pane.
